# Arabic validation and adaptation of the Screen for Cognitive Impairment in Psychiatry (SCIP-A) in university students: a pilot study

**DOI:** 10.3389/fpsyg.2026.1791813

**Published:** 2026-04-15

**Authors:** Mohamed Aly, Helmy Morgan, Turki Alzahrani, Mohammed Fakehy, Abdullah A. Alselaimi, Sheref Mohamed, Scot Purdon

**Affiliations:** 1 Department of Educational Sciences and Sports Psychology, Faculty of Sport Sciences, Assiut University, Assiut, Egypt; 2 Faculty of Liberal Arts and Sciences, Chukyo University, Nagoya, Japan; 3 Department of Sport Sciences, College of Education, Taif University, Taif, Saudi Arabia; 4 Department of Biomechanics and Motor Behavior, College of Sport Sciences and Physical Activity, King Saud University, Riyadh, Saudi Arabia; 5 Department of Psychiatry, University of Alberta, Edmonton, AB, Canada

**Keywords:** Arabic, cognition, cross-cultural adaptation, neuropsychological testing, psychometric properties

## Abstract

**Objective:**

The Screen for Cognitive Impairment in Psychiatry (SCIP) is a brief cognitive screening tool that assesses domains commonly affected in psychiatric populations. Despite its international use, no Arabic version has been available. This study aimed to translate, culturally adapt, and validate the Arabic version (SCIP-A) in a healthy adult sample.

**Methods:**

Following a standardized forward-backward translation and expert committee review, 120 healthy university students (60 male, 60 female) completed the SCIP-A in two sessions 48 h apart, using counterbalanced combinations of its three alternate forms. Psychometric properties were evaluated using Cronbach’s alpha (internal consistency), Intraclass Correlation Coefficients (test–retest reliability), and Confirmatory Factor Analysis (construct validity). A repeated measures ANOVA was also conducted to examine the concurrent effects of time, gender, and alternate forms.

**Results:**

The SCIP-A demonstrated acceptable internal consistency (Cronbach’s *α* = 0.78) and excellent test–retest reliability (ICC = 0.92). Confirmatory factor analysis confirmed an acceptable fit for the proposed two-factor model, supported by a non-significant chi-square test (*χ*^2^ = 10.722, *p* = 0.071) and strong overall fit indices (e.g., CFI = 0.975; SRMR = 0.038). The three alternate forms were found to be equivalent. While minor gender effects were noted on some subtests, performance improvements upon re-administration suggested small practice effects.

**Conclusion:**

The SCIP-A shows sound psychometric properties and form equivalence in a healthy young adult sample. Given its brevity, ease of administration, and availability of alternate forms designed to mitigate practice effects, the SCIP-A appears to be a promising and practical tool for cognitive screening in Arabic-speaking contexts, pending further validation in broader populations.

## 1 Introduction

Cognitive impairments are widely recognized as a fundamental and persistent feature of numerous psychiatric disorders, including schizophrenia (Gebreegziabhere et al., 2022). These deficits span multiple domains—such as attention, verbal and working memory, processing speed, and executive function—and have been consistently linked to poorer functional outcomes (Green et al., 2000), regardless of symptom severity or clinical state (Bora, 2016; Heilbronner et al., 2016). Despite their clinical relevance, the systematic assessment of cognitive functioning remains uncommon in many psychiatric settings, primarily due to practical constraints such as time, cost, and limited access to trained personnel (Purdon, 2005; Miskowiak et al., 2018).

To address this gap, several brief cognitive screening instruments have been developed (Atkinson et al., 2015; Sachs et al., 2024; Dimitriou et al., 2025). However, many were originally designed for older adults or for use in dementia (e.g., MMSE, MoCA) and often lack the sensitivity to detect more subtle cognitive dysfunctions in psychiatric populations (Manning et al., 2007; González-Blanch et al., 2011; Rademeyer and Joubert, 2016). Recent intervention studies underscore the need for tools that can track cognitive changes in psychiatric disorders, particularly in remediation programs (Montejo et al., 2024). Among the few tools specifically developed for psychiatric use, the Screen for Cognitive Impairment in Psychiatry (SCIP) stands out for its brevity, psychometric soundness, and minimal resource requirements. Being performance-based and paper-and-pencil, it can be administered by trained clinical or research staff without requiring specialized neuropsychological expertise, enhancing its practicality in diverse settings. The SCIP assesses five cognitive domains—immediate verbal learning, working memory, verbal fluency, delayed recall, and processing speed—in < 20 min and includes three alternate forms to minimize practice effects (Purdon, 2005). In a direct comparison with the MoCA, Murri et al. (2020) found the SCIP to demonstrate superior psychometric properties and higher diagnostic accuracy in identifying cognitive impairment among patients with psychosis. These findings support its use in both clinical and research contexts, especially in settings where time and resources are limited.

The SCIP has been validated across several languages and populations. Studies conducted in Spain (Pino et al., 2008; Guilera et al., 2009), Italy (Belvederi Murri et al., 2020), Denmark (Ott et al., 2016; Jensen et al., 2021), and Colombia (Castaño Ramírez et al., 2015) have demonstrated its reliability, parallel form equivalence, and construct validity in both clinical and non-clinical samples. Owing to its brevity and parallel forms, the SCIP is suitable for a wide range of settings, including specialist psychiatric services, primary-care triage, community programs, and research applications (Pino et al., 2008; Schmid et al., 2021). It can be applied across diagnostic groups (e.g., schizophrenia, bipolar disorder, major depressive disorder) and at multiple points along the care pathway—from initial screening to baseline characterization and to longitudinal monitoring (Belvederi Murri et al., 2020; Folesani et al., 2022). Collectively, this evidence supports the SCIP as a practical, scalable, and transdiagnostic screener that can guide referral to comprehensive assessment and inform treatment planning in routine practice.

Although the SCIP was originally designed for use in psychiatric populations, growing evidence supports its applicability in healthy individuals as well. Several validation studies have confirmed its reliability and validity in non-clinical samples. For example, the Italian version demonstrated good internal consistency (Cronbach’s *α* = 0.70) and strong test–retest reliability in a sample of 120 healthy adults, with factor analysis revealing a two-factor structure that accounted for 55.4% of the total variance, thereby supporting its structural validity as a cognitive screening tool assessing multiple domains (Belvederi Murri et al., 2020). Similar results were observed in the Spanish version, which showed robust psychometric properties in healthy controls, including high feasibility and strong associations with standard neuropsychological measures (Pino et al., 2008). In a German validation study, the SCIP effectively distinguished between healthy controls and individuals with schizophrenia, performing comparably to other widely used tools such as the Brief Assessment of Cognition in Schizophrenia (BACS) and the Mini-mental state examination (MMSE), and demonstrating strong internal consistency and construct validity (Sachs et al., 2021). Additionally, digital adaptations of the SCIP have also proven effective. The Internet-Based Cognitive Assessment Tool (ICAT), a web-based version of the SCIP, has been tested in healthy samples and shown to have moderate to strong correlations with traditional SCIP scores, indicating strong convergent validity (Hafiz et al., 2019). Usability and feasibility studies of ICAT among non-clinical participants further support its applicability in research and community-based cognitive screening (Hafiz et al., 2019). Taken together, these findings suggest that the SCIP is not only a practical and reliable tool in clinical psychiatry but also a valid instrument for assessing cognitive function in healthy populations, especially when full neuropsychological assessment is not feasible. This need is particularly acute in many Arabic-speaking countries, where the prevalence of severe mental disorders is substantial, yet access to specialized neuropsychological services remains limited (Okasha et al., 2025).

Several validated cognitive instruments exist for Arabic-speaking populations, including the Arabic version of BACS (Haddad et al., 2021). While BACS is practical and widely used, it functions as a fuller brief battery (typically < 35 min) with two alternate forms and was developed primarily for schizophrenia. In contrast, the SCIP was designed as an ultra-brief screen (10–15 min) for cross-screening use and provides three alternate forms that facilitate serial assessments with reduced practice effects. To support scalable cognitive screening in Arabic-speaking contexts, there is a need first to establish the linguistic validity and reliability of SCIP. To our knowledge, no validated Arabic version of the SCIP has been published. Accordingly, we adapted the SCIP into Arabic and evaluated its psychometric properties in a healthy university sample as a foundational step toward later clinical validation.

## 2 Materials and methods

### 2.1 Participants

A total of 120 university students (60 males and 60 females) from Assiut University, aged 20–25 years, voluntarily participated in the study. Recruitment was conducted through institutional email announcements. All participants were native Arabic speakers and provided written informed consent after a clear explanation of the study objectives and procedures. To ensure adequate verbal cognitive functioning, participants completed the Vocabulary subtest of the Wechsler Adult Intelligence Scale—Revised. While specific cutoff scores were not applied due to the lack of validated Arabic norms, this subtest served as a screening tool to confirm sufficient verbal abilities for participation. Additional exclusion criteria included any self-reported history of neurological, psychiatric, or cognitive disorders. Ethical approval for this study was granted by the Institutional Review Board of Assiut University (Approval ID: 102024003).

### 2.2 Instruments

The SCIP is a brief, standardized neurocognitive battery designed to assess five core domains of cognitive functioning commonly affected in psychiatric and neurological populations. The SCIP comprises five subtests: Verbal Learning Test – Immediate (VLT-I), Working Memory Test (WMT), Verbal Fluency Test (VFT), Verbal Learning Test – Delayed (VLT-D), and Psychomotor Speed Test (PST). To minimize learning effects from repeated exposure, the SCIP is available in three equivalent alternate forms (Forms 1, 2, and 3), developed to be structurally and psychometrically comparable (Purdon, 2005). Each subtest in the SCIP targets a distinct cognitive process. The VLT-I assesses verbal learning through three successive presentations of a 10-item word list, with participants recalling as many items as possible after each trial. The WMT evaluates working memory using eight sequences of three unrelated consonants, which must be recalled either immediately or following a brief distraction task involving backward counting. The VFT measures verbal fluency by asking participants to generate as many words as possible beginning with specific letters under time constraints, excluding proper nouns and repetitions. The VLT-D examines delayed verbal recall by prompting participants to retrieve the word list from the VLT-I after a delay, without any re-presentation. Finally, the PST assesses processing speed and visual-motor coordination; participants are shown a reference key linking letters to Morse code and are asked to reproduce the corresponding symbols for a series of letters within a set time, following a short practice phase. A total score is computed by summing the raw scores from all subtests, with higher scores indicating better cognitive functioning and a total score ≥ 70 has been suggested to correspond to normal cognitive functioning (Rojo et al., 2010).

**Figure 1:**
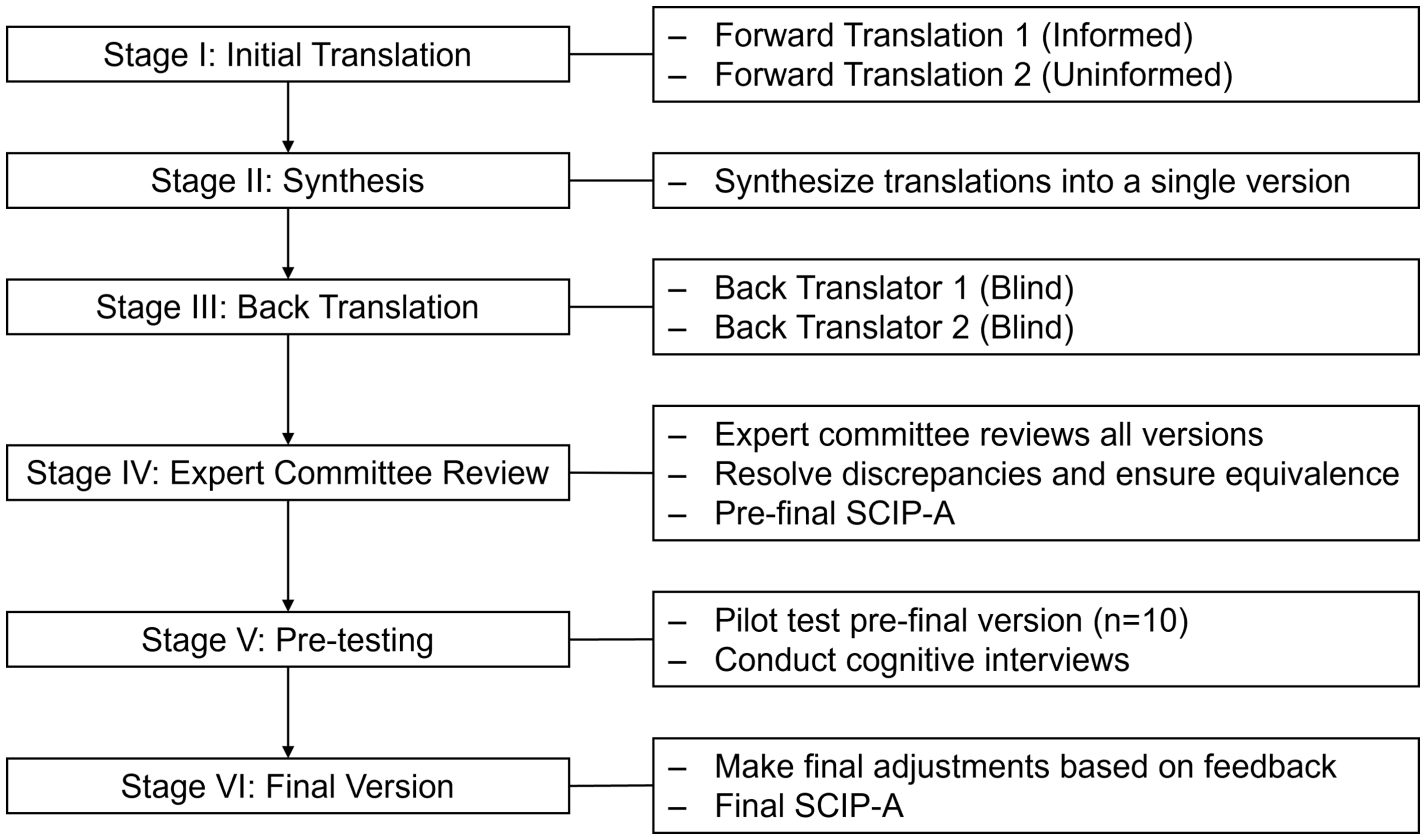
Translation and validation process of the SCIP-A.

The translation and cross-cultural adaptation of the SCIP-A followed the established multi-stage guidelines proposed by Beaton et al. (2000). Stages I–II (forward translation and synthesis) were conducted first. Two independent forward translations from English to Arabic were produced by professional translators who were native Arabic speakers fluent in English—one informed about the test’s concepts and the other uninformed. A synthesized version was created from these two translations. Next, in Stage III (Back Translation), two additional translators, native English speakers with no prior knowledge of the SCIP, independently back-translated the synthesized Arabic version into English. For Stage IV (Expert Committee), an expert committee, consisting of the study authors, two bilingual clinical psychologists, and a linguist, reviewed all translations to identify and resolve any semantic, idiomatic, or conceptual discrepancies. Disagreements were resolved through iterative discussion until a consensus was reached, resulting in a pre-final version of the SCIP-A. Finally, in Stage V (Pre-testing), this pre-final version was pilot tested on a sample of 10 university students representative of the target population. During this pilot phase, informal cognitive interviews were conducted with 10 university students. Participants were asked to ‘think aloud’ as they reviewed the instructions and items to ensure their interpretation matched the original intent. Feedback confirmed the comprehensibility of most items, but the pre-testing phase was critical in validating several key linguistic adaptations that had been proposed by the expert committee. These included: (1) Phonetic Replacements in the VFT and WMT, where English letters without direct Arabic phonetic equivalents (e.g., ‘C’, ‘P’, ‘X’) were replaced with unambiguous Arabic consonants (e.g., ‘ص’, ‘ع’, ‘ث’); (2) Lexical Replacements in the Verbal Learning Test (VLT), where specific words were substituted for cultural or frequency equivalence (e.g., ‘Doll’ was replaced with ‘تمثال’ [Statue] in Form 2); and (3) Directional Changes in the Processing Speed Test (PST), where the instructions were modified from ‘left-to-right’ to ‘right-to-left’ to match Arabic reading conventions. Feedback from the cognitive interviews confirmed these changes felt natural and were clearly understood. A summary of these key adaptations is provided in Table 1. The entire multi-stage adaptation process is summarized in Figure 1.

**Table 1:** Summary of key linguistic and cultural adaptations in the SCIP-A.

Subtest	Adaptation type	Original english stimuli/instruction	Final Arabic (SCIP-A) adaptation	Rationale
Verbal Fluency (VFT) - Form 1	Phonetic Replacement	Letter: ‘C’	Letter: ‘ث’ (Th)	The English letter ‘C’ is phonetically ambiguous and has no direct Arabic equivalent. ‘ث’ is an unambiguous Arabic consonant.
Verbal Fluency (VFT) - Form 2	Phonetic Replacement	Letter: ‘P’	Letter: ‘ع’ (Ayn)	The /p/ sound does not exist in standard Arabic. ‘ع’ is a unique and unambiguous Arabic consonant.
Working Memory (WMT) Form 1	Phonetic Replacement	Consonant String: Q-L-X	Consonant String: ق - ل - ص (Q-L-S)	The letter ‘X’ (/ks/) is not a standard Arabic phoneme. It was consistently replaced with phonetically clear consonants like ‘ص’ (S) or ‘ث’ (Th).
Verbal Learning (VLT-I) - Form 2	Lexical Replacement	Word: ‘Doll’	Word: ‘تمثال’ (Statue)	Substitution to ensure comparable word frequency and cultural familiarity for the Arabic-speaking sample.
Verbal Learning (VLT-I) - Form 2	Lexical Replacement	Word: ‘Mirror’	Word: ‘مسبح’ (Pool)	Substitution for cultural/linguistic relevance, confirmed as clear and appropriate during cognitive interviews.
Processing Speed (PST) - All Forms	Directional Adaptation	Instruction: “…complete left to right…”	Instruction: “إبدا الاختبار من اليمين إلى اليسار” (Start the test from right to left)	To match the standard reading and writing direction of the Arabic language.

### 2.3 Procedure

Participants completed two testing sessions, beginning with the administration of the WAIS-R Vocabulary subtest followed by one of the three alternate forms of the Arabic version of the Screen for Cognitive Impairment in Psychiatry (SCIP-A). Participants were evenly assigned to one of the three forms (n = 40 per form) during the initial session. The second testing session occurred after a short interval 48-h delay, with a mean deviation of −15 min (SD = 85 min), during which participants were administered the second form of the SCIP-A. We selected a short retest interval to minimize true change in a healthy, nonclinical cohort while allowing us to estimate temporal reliability and practice effects separately using alternate forms. To ensure counterbalancing between the alternate forms, participants were further divided into six subgroups (n = 20 per subgroup), with equal gender distribution within each. Each subgroup was assigned a specific sequence of forms to allow for evaluation of alternate-form reliability: Subgroup AC received Forms 1 and 2, Subgroup BE received Forms 1 and 3, and Subgroup DF received Forms 2 and 3. The order of form administration was also counterbalanced within each subgroup (e.g., half of participants received Form 1 first and Form 2 s, while the other half received Form 2 first and Form 1 s), enabling thorough analysis of test–retest and parallel form equivalence. Supplementary Table S1 provides further illustration of the alternate form pairings and administration order.

### 2.4 Statistical analyses

The reliability of the SCIP-A scores was initially assessed by calculating internal consistency using Cronbach’s alpha. To evaluate test–retest reliability, we calculated Intraclass Correlation Coefficients (ICC) between subtest scores obtained at Time 1 and Time 2. Values less than 0.5, between 0.5 and 0.75, between 0.75 and 0.9, and greater than 0.90 are indicative of poor, moderate, good, and excellent reliability, respectively (Koo and Li, 2016). In order to explore factors that may influence SCIP-A scores (practice effects, effect of alternate forms, and gender), we conducted a repeated measures ANOVA with time as a within subject variable (time 1 vs. time 2), gender (male vs. female) and subgroup receiving alternate forms (AC vs. BE vs. DF), as between subject variables.

A confirmatory factor analysis (CFA) was conducted on the total sample to confirm two-factor pattern—WMT, VFT, VLT-D loading on Factor 1 and VLT-I, PST on Factor 2 specified *a priori* from the Italian validation (Belvederi Murri et al., 2020). Maximum Likelihood was used as the method of estimation. The following fit indices were calculated to check the adequacy of the model: the chi-square test (*χ*^2^), Comparative Fit Index (CFI), Tucker–Lewis Index (TLI), Root Mean Square Error of Approximation (RMSEA), and Standardized Root Mean Square Residual (SRMR). Values ≤ 0.05 for SRMR, ≤ 0.08 for RMSEA, and ≥ 0.90 for CFI and TLI indicate good fit of the model to the data (Hu and Bentler, 1999).

Alpha level was set at the conventional value of 0.05 for determining statistical significance, and partial eta-squared (η_p_^2^) was calculated to describe effect sizes. SPSS 25.0 (IBM, Armonk, NY) was used for all analyses except CFA that we utilized the lavaan package in r Studio (Rosseel, 2012).

## 3 Results

Descriptive characteristics of the sample are presented in Table 2. Complete data were obtained from all participants.

**Table 2:** Description of the sample (*n* = 120).

Variable	Mean	SD
Gender, female (*n* = 60, 50%)		
Age	21.33	0.82
Years of education	14.30	0.74
WAIS-R vocabulary adj score	11.57	2.58

### 3.1 Reliability and correlates

Internal consistency for the SCIP-A total score was acceptable (Cronbach’s *α* = 0.78). As recommended for multidimensional scales, internal consistency was also calculated for the two factors. The Cronbach’s alpha was acceptable at 0.74 for the three-item ‘working memory and verbal fluency’ factor, and 0.63 for the two-item ‘learning and processing speed’ factor. The lower alpha for the second factor is likely attributable to the small number of items contributing to that subscale.

Test–retest reliability was evaluated by calculating the ICC between scores at Time 1 and Time 2. The total SCIP-A score demonstrated excellent stability over time (ICC = 0.92). Subtest ICCs ranged from 0.77 for the VFT to 0.89 for the VLT-I, indicating good to excellent reliability across all domains (see Table 3, upper panel).

**Table 3:** Raw values and correlation between SCIP subtests and total score at time 1 and time 2.

Subtest	Time 1	Time 2	ICC (95% CI)
	Mean (SD)	Mean (SD)	
VLT-I	24.65 (3.27)	25.28 (3.20)	0.89 (0.81–0.94)
WMT	21.23 (3.20)	21.83 (3.10)	0.86 (0.78–0.91)
VFT	18.30 (1.97)	18.32 (2.37)	0.77 (0.68–0.83)
VLT-D	8.38 (1.47)	8.43 (1.48)	0.84 (0.77–0.88)
PST	13.00 (4.84)	13.69 (4.26)	0.80 (0.72–0.86)
Total SCIP	85.57 (11.57)	87.56 (11.67)	0.92 (0.86–0.95)

Subtest	AC (form 1–2 and 2–1)	BE (form 1–3 and 3–1)	DF (form 2–3 and 3–2)
	ICC (95% CI)	ICC (95% CI)	ICC (95% CI)
VLT-I	0.91 (0.80–0.96)	0.86 (0.75–0.92)	0.91 (0.61–0.97)
WMT	0.60 (0.28–0.78)	0.87 (0.77–0.93)	0.93 (0.86–0.96)
VFT	0.75 (0.58–0.86)	0.61 (0.37–0.77)	0.91 (0.83–0.95)
VLT-D	0.85 (0.71–0.92)	0.71 (0.51–0.83)	0.93 (0.88–0.97)
PST	0.75 (0.58–0.86)	0.81 (0.67–0.90)	0.85 (0.73–0.92)
Total SCIP	0.90 (0.74–0.96)	0.89 (0.81–0.94)	0.96 (0.82–0.99)

VLT-I, Verbal Learning Test-Immediate; WMT, Working Memory Test; VFT, Verbal Fluency Test; VLT-D, Verbal Learning Test-Delayed; PST, psychomotor speed test.

To further assess the reliability across alternate test forms, ICCs were computed for each subgroup (AC, BE, and DF). Overall, the alternate forms showed strong reliability, particularly for the Total SCIP score (subgroup ICCs ranged from 0.89 to 0.96). However, some variability was observed at the subtest level. For example, the DF group (forms 2 and 3) demonstrated the most consistent high reliability across subtests (ICCs ≥ 0.91), while the AC and BE groups showed lower reliability for the WMT and VFT subtests, respectively (see Table 3, lower panel).

Gender differences were observed at the subtest level, with males performing significantly better than females on the WMT (*p* = 0.014; Hedges’ g = 0.456), while females outperformed males on the Verbal Learning Test–Immediate (VLT-I; *p* = 0.033; Hedges’ g = 0.391). No other gender-based differences reached statistical significance (see Supplementary Table S2). Additionally, no significant differences were found in SCIP-A total or subtest scores across the three alternate forms at Time 1, suggesting form equivalence at baseline (all *p* > 0.10; see Supplementary Table S3). Raw mean scores by gender, alternate form group, and time point are presented in Supplementary Table S4.

### 3.2 Concurrent effects of time, gender and alternate forms

A series of repeated measures two-way ANOVAs was conducted to examine the effects of time, gender, and alternate test forms on SCIP-A subtest and total scores (see Table 4). For VLT-I, there was a significant main effect of time (*p* < 0.001, η_p_^2^ = 0.182, a large effect), indicating improved scores from Time 1 to Time 2. A significant effect of gender was also observed (*p* = 0.033, η_p_^2^ = 0.038, a small effect), with females scoring higher than males. For WMT, significant effects were found for both time (*p* < 0.001, η_p_^2^ = 0.139, a large effect) and gender (*p* = 0.038, η^2^ = 0.037, a small effect), indicating performance gains over time and higher scores among males. VFT and VLT-D did not show any significant main or interaction effects. PST showed a significant effect of time (*p* = 0.010, η_p_^2^ = 0.055, a small-to-medium effect), with improved scores at Time 2, but no significant effects of gender or test form. Finally, the total SCIP-A score revealed a strong effect of time (*p* < 0.001, η_p_^2^ = 0.194, a large effect), reflecting overall performance improvement. No significant main effects or interactions involving test form were detected across any of the subtests or the total score, indicating equivalence of alternate forms.

**Table 4:** Repeated measures two-way ANOVA.

Subtests		df	MS	*F*	*p*	*ɳ* ^2^
VLT-I						
Within subject	Time**	1	23.438	25.320	< 0.001	0.182
	Time × forms	2	1.163	1.256	0.289	0.022
	Time × gender	1	0.204	0.221	0.640	0.002
	Time × forms × gender	2	2.004	2.165	0.119	0.037
Between subject	Forms	2	33.538	1.793	0.171	0.030
	Gender*	1	87.604	4.683	0.033	0.039
	Forms × gender	2	44.879	2.399	0.095	0.040
WMT						
Within subject	Time**	1	21.600	18.349	< 0.001	0.139
	Time × forms	2	2.150	1.826	0.166	0.031
	Time × gender	1	4.267	3.624	0.059	0.031
	Time × forms × gender	2	0.817	0.694	0.502	0.012
Between subject	Forms	2	9.867	0.532	0.589	0.009
	Gender*	1	81.667	4.401	0.038	0.037
	Forms × gender	2	1.517	0.082	0.922	0.001
VFT						
Within subject	Time	1	0.017	0.016	0.901	0.000
	Time × forms	2	3.004	2.823	0.064	0.047
	Time × gender	1	2.400	2.256	0.136	0.019
	Time × forms × gender	2	1.138	1.069	0.347	0.018
Between subject	Forms	2	5.579	0.660	0.519	0.011
	Gender	1	6.667	0.789	0.376	0.007
	Forms × gender	2	6.979	0.826	0.440	0.014
VLT-D						
Within subject	Time	1	0.150	0.425	0.516	0.004
	Time × forms	2	0.988	2.800	0.065	0.047
	Time × gender	1	0.150	0.425	0.516	0.004
	Time × forms × gender	2	0.263	0.744	0.477	0.013
Between subject	Forms	2	0.879	0.222	0.801	0.004
	Gender	1	7.350	1.855	0.176	0.016
	Forms × gender	2	6.088	1.536	0.220	0.026
PST						
Within subject	Time*	1	28.704	6.847	0.010	0.057
	Time × forms	2	2.279	0.544	0.582	0.009
	Time × gender	1	0.504	0.120	0.729	0.001
	Time × forms × gender	2	0.904	0.216	0.806	0.004
Between subject	Forms	2	14.904	0.387	0.680	0.007
	Gender	1	14.504	0.376	0.541	0.003
	Forms × gender	2	9.629	0.250	0.779	0.004
Total SCIP						
Within subject	Time**	1	238.004	27.447	< 0.001	0.194
	Time × forms	2	13.679	1.578	0.211	0.027
	Time × gender	1	7.704	0.888	0.348	0.008
	Time × forms × gender	2	8.954	1.033	0.359	0.018
Between subject	Forms	2	187.363	0.705	0.496	0.012
	Gender	1	78.204	0.294	0.589	0.003
	Forms × gender	2	180.104	0.678	0.510	0.012

VLT-I, Verbal Learning Test-Immediate; WMT, Working Memory Test; VFT, Verbal Fluency Test; VLT-D, Verbal Learning Test-Delayed; PST, psychomotor speed test.

^*^p < 0.05, ^**^p < 0.01.

### 3.3 Confirmatory factor analysis (CFA) of the SCIP-A

The CFA results, shown in Table 5, indicated an acceptable fit for the proposed two-factor model. The chi-square test was non-significant (*χ*^2^ = 10.722, *p* = 0.071), suggesting no significant discrepancy between the model and the observed data. Furthermore, other fit indices were excellent, including the CFI (0.975), TLI (0.950), and SRMR (0.038). Although the RMSEA point estimate was 0.098, its wide 90% confidence interval [0.000–0.179] includes values indicative of a close fit, suggesting imprecision in its estimate. Given the strong support from the majority of fit indices and the non-significant chi-square test, the model was deemed an acceptable representation of the data.

**Table 5:** Standardized factor loadings and model fit for the two-factor CFA model.

Factor	Subtest	Standardized loading (β)
Working memory & verbal fluency	WMT	0.692
	VFT	0.729
	VLT-D	0.634
Learning & processing speed	VLT-I	0.871
	PST	0.672

VLT-I, Verbal Learning Test-Immediate; WMT, Working Memory Test; VFT, Verbal Fluency Test; VLT-D, Verbal Learning Test-Delayed; PST, psychomotor speed test.

All factor loadings were statistically significant (*p* < 0.001). Model Fit Indices: Chi-square (Bollen–Stine) = 10.722, *p* = 0.071; CFI = 0.975; TLI = 0.950; RMSEA = 0.098 (CI: 0.000–0.179); SRMR = 0.038.

All individual subtests loaded significantly on their respective latent factors. The standardized factor loadings were moderate to very strong, ranging from 0.634 to 0.871. Specifically, the loadings were as follows: VLT-I = 0.692, WMT = 0.729, VFT = 0.634, VLT-D = 0.871, and PST = 0.672. These results provide strong support for the construct validity of the proposed two-factor structure of the SCIP-A in the university student sample.

## 4 Discussion

To our knowledge, this is the first study to validate the Arabic version of SCIP, providing foundational evidence for its use as a brief neuropsychological screening tool for cognitive assessment in young, educated Arabic-speaking adults. The results demonstrated that the SCIP-A possesses sound psychometric properties. Internal consistency was acceptable for its two primary factors, test–retest reliability was excellent, and the instrument showed strong construct validity and parallel form equivalence. The normative performance observed in our sample was comparable to findings from previous international validation studies. These results offer encouraging evidence for the cross-cultural applicability of the SCIP and support its potential use in both research and clinical practice within Arabic-speaking settings.

The SCIP-A includes three alternate forms designed to assess neuropsychological functioning across repeated sessions while minimizing practice effects. In the present study, we tested all three forms and found no significant differences in total scores or subtest scores at baseline, supporting their equivalence. These findings are consistent with those reported in earlier validation studies, including the original Canadian version (Purdon, 2005), the Spanish version (Pino et al., 2006), and the Italian adaptation (Belvederi Murri et al., 2020), which also demonstrated minimal or no effects of form. The observed consistency suggests that the alternate forms of the SCIP-A can be used interchangeably in Arabic-speaking populations, facilitating reliable longitudinal assessment.

Acceptable test–retest reliability was observed across all SCIP-A subtests in our sample, with excellent reliability for the total score and good to excellent reliability coefficients for the individual subtests. These findings are consistent with previous reports supporting the temporal stability of the SCIP-A (Purdon, 2005; Pino et al., 2006). Despite the use of alternate forms, we observed evidence of practice effects, particularly for the total score, working memory, and processing speed subtests. Scores generally improved from Time 1 to Time 2, suggesting that repeated exposure may influence performance, especially in tests with procedural or speeded components. This pattern aligns with earlier studies, which reported similar improvements upon re-administration (Purdon, 2005; Pino et al., 2006). While the use of alternate forms may reduce such effects to some extent, they do not fully eliminate such effects. Therefore, when using the SCIP-A in repeated measurements, especially over short intervals, it is important to account for potential learning effects to avoid overestimating treatment-related gains or changes in cognitive status. For researchers conducting clinical trials, this highlights the importance of including a control group to help disentangle true cognitive improvements from practice effects. For clinicians using the SCIP-A for longitudinal monitoring, it suggests that small improvements in scores upon re-testing over a short interval may reflect procedural learning rather than a clinically significant change in cognitive function. The mean performance gain on the total score in our non-clinical sample (approximately 2 points) can serve as a preliminary, conservative benchmark for estimating these effects. Based on this, longer re-assessment intervals are advisable for longitudinal monitoring.

Gender showed a modest but statistically significant influence on specific SCIP-A subtest scores in our sample. At Time 1, males outperformed females on the WMT, while females performed better than males on the VLT-I. No significant gender differences were found for the total SCIP-A score, and no interactions were observed between gender and test form or between time and gender for the total score, indicating that cognitive changes over time were similar for both sexes. These findings are partially consistent with those from previous studies. In the Italian validation, Belvederi Murri et al. (2020) reported gender-related effects in WMT and PST, with women outperforming men in VLT-I and PST at baseline, and men showing superior performance in WMT at follow-up. Similarly, the original manual by Purdon (2005) reported that men performed better than women on WMT at baseline, whereas women outperformed men in PST and VLT-D during retesting. Pino et al. (2006), in the Spanish version of the SCIP, found that gender explained 11% of the total variance, with men scoring higher in PST. Although the patterns vary across studies and populations, these recurring findings suggest that gender may influence specific cognitive domains assessed by the SCIP. Accordingly, consideration of participant sex may enhance the interpretability of subtest scores in both clinical and research applications.

Our analysis of the SCIP-A’s internal structure provided evidence for its construct validity. A CFA testing the *a priori* two-factor showed an acceptable overall model fit and moderate to very strong, significant factor loadings. This supports a structure that, in line with the Italian validation, differentiates a factor comprising Working Memory, Verbal Fluency, and Delayed Verbal Learning from a second factor comprising Immediate Verbal Learning and Processing Speed. This pattern aligns with findings from previous Spanish and Italian validations (Pino et al., 2006; Belvederi Murri et al., 2020), suggesting cross-cultural stability of the instrument’s underlying constructs. The clinical relevance of these domains is highlighted by the fact that they are often impaired in disorders such as schizophrenia and bipolar disorder (González-Blanch et al., 2011; Bora, 2016; Murri et al., 2020), supporting the SCIP-A’s utility in clinical research. In contrast, studies conducted in patient populations (Pino et al., 2008; Guilera et al., 2009) have often identified a single-factor solution, likely reflecting reduced variability across subtests in cognitively impaired samples. Together, these findings suggest that the SCIP-A captures distinct but related cognitive domains in healthy individuals, whereas performance may converge in clinical contexts due to generalized impairment.

The validation of the SCIP-A has several important implications for mental healthcare in Arabic-speaking regions. For clinicians, it provides a rapid, reliable, and resource-efficient tool to integrate cognitive screening into routine psychiatric practice, aiding in early detection, treatment planning, and monitoring of cognitive changes in busy clinical settings where a full neuropsychological workup is not feasible (Miskowiak et al., 2018). For researchers, the SCIP-A opens new avenues for investigation by helping to overcome the well-documented methodological and cross-cultural barriers in neuropsychological assessment (Veliu and Leathem, 2017). It enables robust cross-cultural studies and facilitates clinical trials with cognitive outcomes for Arabic-speaking populations. For policymakers, the availability of a validated tool can support wider public health initiatives and inform clinical care guidelines that include cognitive assessment as a standard of care (Saxena et al., 2014). Specifically, in busy primary care or psychiatric outpatient settings, its brevity (< 20 min) makes it a feasible tool for initial screening at intake or for annually monitoring cognitive health, helping to triage patients who may require referral to a neuropsychologist. However, clinicians must exercise caution when interpreting scores from older individuals or those with different educational backgrounds, as the current normative data are derived from young, educated adults.

The present findings offer preliminary support for the use of the Arabic version of the SCIP as a reliable and practical cognitive screening tool in Arabic-speaking settings. Indeed, a direct comparison of normative performance highlights this cross-cultural consistency. The mean total score in our Arabic-speaking sample (*M* = 85.57) is remarkably similar to that reported in the Italian validation study (*M* = 86.02; Belvederi Murri et al., 2020), which also used a university student sample. Both scores are, as expected, slightly higher than those from an older Spanish normative sample (*M* = 81.33; Pino et al., 2008), demonstrating a consistent pattern across language versions. The high prevalence of adverse childhood experiences (ACEs) among individuals with schizophrenia (Assefa Fentahun et al., 2024) further emphasizes the need for culturally adapted cognitive screening tools like the SCIP-A to identify at-risk individuals early. Given its brevity, ease of administration, and inclusion of alternate forms to mitigate retest effects, the SCIP-A is particularly well-suited for use in busy clinical environments where time and resources may be limited. The two-factor structure identified in this study reinforces the tool’s ability to capture distinct cognitive domains—namely verbal memory and processing speed—which are often affected in psychiatric and neurological conditions. Importantly, there is a notable paucity of validated cognitive assessment tools available in the Arabic language, which poses a challenge for clinicians and researchers working with Arabic-speaking populations (Aly et al., 2024b). This study contributes to filling that gap by providing a culturally adapted and validated tool that is not only suitable for clinical applications but also for use with healthy populations (Purdon, 2005; Pino et al., 2008; Belvederi Murri et al., 2020). It addresses the clear need in the Arab world for instruments that can replicate prior research examining the effects of behavioral interventions aimed at enhancing or maintaining cognitive functioning (Aly et al., 2019, 2023, 2024a,c,d, 2025). The availability of preliminary performance data from a healthy young Arabic-speaking sample further enhances its utility as an initial reference point for future clinical evaluations, early detection of cognitive impairment, and longitudinal monitoring of cognitive change.

Despite the promising psychometric properties observed in this study, several limitations should be acknowledged. First, the sample consisted exclusively of healthy adult participants, which limits the generalizability of the findings to clinical populations. A direct consequence of this design is that it was not possible to establish a clinical cut-off score for identifying cognitive impairment. Future research in clinical populations is therefore essential to both evaluate the SCIP-A’s performance in patient groups and to determine these crucial screening thresholds. Second, although test–retest reliability was assessed, the retest interval was relatively short (i.e., 48 h), which may have increased susceptibility to practice effects. While the use of alternate forms was intended to mitigate this issue, it did not entirely eliminate performance gains between assessments. Moreover, a longer interval between administrations would more accurately reflect real-world clinical practice, where cognitive reassessments are typically conducted after several months or even a year (Beglinger et al., 2014; Feeney et al., 2016). Third, our participants were university students aged 19 to 25 years, representing a relatively narrow age range and a high level of education. Crucially, this sample homogeneity limits the generalizability of our findings to the entire Arabic-speaking population, especially those with limited or no formal education. Specifically, while we observed statistically significant gender differences on certain subtests, these results must be interpreted with caution. They reflect performance within a young, highly educated cohort and may not be applicable to the broader Arabic-speaking population, which encompasses a wider range of ages, literacy levels, and educational backgrounds. Further studies in more diverse samples are essential to establish comprehensive norms and validate these preliminary findings. Fourth, while the internal consistency of the first factor was acceptable, the alpha for the second factor (‘learning and processing speed’) was low. This is likely a statistical artifact due to the factor being composed of only two subtests, as Cronbach’s alpha is sensitive to the number of items in a scale. Nevertheless, this suggests that scores derived from this second factor should be interpreted with more caution than the total score or the first factor score. Fifth, regarding our factor analysis, the chosen two-factor model demonstrated an acceptable fit, though it should be interpreted with some caution. While most indices (non-significant *χ*^2^, CFI, TLI, SRMR) strongly supported the model, the RMSEA value was elevated above conventional thresholds. However, the wide confidence interval for the RMSEA suggests its point estimate was imprecise and that a close fit remains plausible. We also note that a more parsimonious single-factor model showed a superior statistical fit. Despite this, we retained the two-factor model because it is theoretically consistent with previous international validations of the SCIP in healthy samples and offers a more nuanced, multidimensional view of the cognitive constructs being measured. Finally, while this study establishes the psychometric foundation of the SCIP-A, several broader cultural factors warrant consideration for its real-world application. Our findings showed cross-cultural comparability, but performance on neuropsychological tests can be influenced by factors beyond simple translation. For instance, the quality and style of education, not just the number of years, can impact test-taking skills and may vary across the Arabic-speaking world. Consequently, the tool cannot yet be fully deployed across the entire population without further validation; while our sample was fully literate, the SCIP-A’s performance in populations with lower literacy rates or those who are uneducated is unknown. Furthermore, the Arabic language has significant regional and dialectal variations. Although our adaptation aimed for a standard form of Arabic, the influence of regional dialects on the interpretation of instructions or on performance in verbal tasks (e.g., VFT) was not systematically examined. These factors should be carefully considered when interpreting SCIP-A scores in different contexts. Therefore, future studies incorporating a representative sample of the uneducated population are essential to explore the applicability of the SCIP-A in more diverse subgroups, such as older adults, individuals with low literacy, and specific clinical populations across different Arab countries.

## 5 Conclusion

This study presents the first validation of the Arabic version of the SCIP, offering a brief and accessible instrument for assessing cognitive functioning, with this initial validation conducted in a sample of young, educated Arabic-speaking adults. The tool demonstrated solid psychometric properties, including strong internal consistency, test–retest reliability, and a meaningful two-factor structure. Additionally, the use of alternate forms proved largely equivalent. While not eliminating practice effects entirely, these forms are a key feature for longitudinal assessment, with minimal confounding effects related to gender or repeated testing. The SCIP-A’s brevity, simple administration, and minimal resource requirements enhance its suitability for use in both research and clinical settings, particularly where time and capacity for extensive assessments are limited. By addressing the gap in validated Arabic-language cognitive screening tools, this study contributes a valuable resource for the early detection of cognitive difficulties. Although not intended for diagnostic purposes, the SCIP-A may serve as a useful preliminary tool to flag individuals who could benefit from further, more comprehensive neuropsychological evaluation.

## Data Availability

The raw data supporting the conclusions of this article will be made available by the authors, without undue reservation.
