# Supplementary material for: Arabic validation and adaptation of the Screen for Cognitive Impairment in Psychiatry (SCIP-A) in university students: a pilot study
Source: Front Psychol. 2026 Apr 15;17:1791813. doi: 10.3389/fpsyg.2026.1791813 (PMC13124518; doi:10.3389/fpsyg.2026.1791813)
Supplement: Supplementary file 1 [file Table_1.DOCX]

Supplementary Tables

**Table S1.** SCIP versions administered at the first and the second times (with a 48-hour delay) to the six sub-groups (n = 20)

| Sub-group | SCIP alternate form | |
| --- | --- | --- |
|  | Time 1 | Time 2 |
| A | 1 | 2 |
| B | 1 | 3 |
| C | 2 | 1 |
| D | 2 | 3 |
| E | 3 | 1 |
| F | 3 | 2 |

In further analysis, these subgroups were joined to account for the alternate forms received by participants: AC (forms 1 and 2), BE (forms 1 and 3), and DF (forms 2 and 3)

**Table S2.** Mean SCIP scores by gender at time 1

| Subtest | Males | | |  | Females | | |  | Significance | | |
| --- | --- | --- | --- | --- | --- | --- | --- | --- | --- | --- | --- |
|  | N | Mean | SD |  | N | Mean | SD |  | *p* | *t* | Hedges g |
| VLT-I | 60 | 24.02 | 2.914 |  | 60 | 25.28 | 3.503 |  | 0.033 | -2.153 | 0.391 |
| WMT | 60 | 21.95 | 2.752 |  | 60 | 20.52 | 3.476 |  | 0.014 | 2.504 | 0.456 |
| VFT | 60 | 18.57 | 1.826 |  | 60 | 18.03 | 2.083 |  | 0.139 | 1.491 | 0.276 |
| VLT-D | 60 | 8.58 | 1.293 |  | 60 | 8.18 | 1.610 |  | 0.136 | 1.500 | 0.274 |
| PST | 60 | 13.20 | 5.048 |  | 60 | 12.80 | 4.653 |  | 0.653 | 0.451 | 0.082 |
| Total SCIP | 60 | 86.32 | 10.771 |  | 60 | 84.817 | 12.369 |  | 0.480 | 0.708 | 0.130 |

**Table S3.** Mean SCIP scores by alternate forms at time 1

| Subtest | Form 1 | | |  | Form 2 | | |  | Form 3 | | |  | Significance | |
| --- | --- | --- | --- | --- | --- | --- | --- | --- | --- | --- | --- | --- | --- | --- |
|  | N | Mean | SD |  | N | Mean | SD |  | N | Mean | SD |  | *f* | *p* |
| VLT-I | 40 | 25.1 | 3.9 |  | 40 | 25.1 | 3.1 |  | 40 | 23.8 | 2.6 |  | 1.939 | 0.148 |
| WMT | 40 | 21.4 | 2.1 |  | 40 | 21.4 | 3.1 |  | 40 | 20.9 | 4.2 |  | 0.280 | 0.756 |
| VFT | 40 | 18.8 | 2.1 |  | 40 | 18.1 | 1.7 |  | 40 | 18.0 | 2.0 |  | 2.177 | 0.118 |
| VLT-D | 40 | 8.3 | 1.6 |  | 40 | 8.5 | 1.3 |  | 40 | 8.3 | 1.5 |  | 0.279 | 0.757 |
| PST | 40 | 13.3 | 5.4 |  | 40 | 12.8 | 4.7 |  | 40 | 13.0 | 4.5 |  | 0.117 | 0.890 |
| Total SCIP | 40 | 86.9 | 11.0 |  | 40 | 85.7 | 11.1 |  | 40 | 84.1 | 12.6 |  | 0.619 | 0.540 |

**Table S4.** Raw mean SCIP scores by gender, alternate forms and time

|  |  |  |  | VLT-I | | WMT | | VFT | | VLT-D | | PST | | Tot | |
| --- | --- | --- | --- | --- | --- | --- | --- | --- | --- | --- | --- | --- | --- | --- | --- |
|  |  |  | N | *M* | *SD* | *M* | *SD* | *M* | *SD* | *M* | *SD* | *M* | *SD* | *M* | *SD* |
| Time 1 | AC | M | 20 | 24.85 | 3.28 | 22.10 | 2.00 | 19.10 | 2.17 | 8.60 | 1.50 | 13.75 | 6.02 | 88.40 | 10.42 |
|  |  | F | 20 | 25.30 | 4.46 | 20.75 | 1.94 | 18.55 | 2.11 | 8.05 | 1.67 | 12.80 | 4.85 | 85.45 | 11.67 |
|  |  |  | 40 | 25.08 | 3.87 | 21.43 | 2.06 | 18.83 | 2.14 | 8.33 | 1.59 | 13.28 | 5.42 | 86.93 | 11.02 |
|  | DF | M | 20 | 23.40 | 2.76 | 22.10 | 2.29 | 18.10 | 1.62 | 8.45 | 1.28 | 12.50 | 4.80 | 84.55 | 10.72 |
|  |  | F | 20 | 26.70 | 2.56 | 20.60 | 3.56 | 18.00 | 1.86 | 8.60 | 1.31 | 13.00 | 4.68 | 86.90 | 11.65 |
|  |  |  | 40 | 25.05 | 3.11 | 21.35 | 3.05 | 18.05 | 1.72 | 8.53 | 1.28 | 12.75 | 4.68 | 85.73 | 11.12 |
|  | BE | M | 20 | 23.80 | 2.61 | 21.65 | 3.76 | 18.50 | 1.57 | 8.70 | 1.13 | 13.35 | 4.37 | 86.00 | 11.34 |
|  |  | F | 20 | 23.85 | 2.72 | 20.20 | 4.57 | 17.55 | 2.24 | 7.90 | 1.80 | 12.60 | 4.66 | 82.10 | 13.80 |
|  |  |  | 40 | 23.83 | 2.63 | 20.93 | 4.20 | 18.03 | 1.97 | 8.30 | 1.54 | 12.98 | 4.47 | 84.05 | 12.62 |
|  | Total | M | 60 | 24.02 | 2.91 | 21.95 | 2.75 | 18.57 | 1.83 | 8.58 | 1.29 | 13.20 | 5.05 | 86.32 | 10.77 |
|  |  | F | 60 | 25.28 | 3.50 | 20.52 | 3.48 | 18.03 | 2.08 | 8.18 | 1.61 | 12.80 | 4.65 | 84.82 | 12.37 |
|  |  | Tot | 120 | 24.65 | 3.27 | 21.23 | 3.20 | 18.30 | 1.97 | 8.38 | 1.47 | 13.00 | 4.84 | 85.57 | 11.57 |
| Time 2 | AC | M | 20 | 25.35 | 3.13 | 22.55 | 2.01 | 18.25 | 2.57 | 8.80 | 1.01 | 14.95 | 5.22 | 89.90 | 11.00 |
|  |  | F | 20 | 26.25 | 3.55 | 22.20 | 1.85 | 18.55 | 2.31 | 8.50 | 1.43 | 13.65 | 4.18 | 89.15 | 10.44 |
|  |  |  | 40 | 25.80 | 3.34 | 22.38 | 1.92 | 18.40 | 2.42 | 8.65 | 1.23 | 14.30 | 4.71 | 89.53 | 10.59 |
|  | DF | M | 20 | 24.15 | 3.30 | 22.25 | 1.59 | 18.05 | 2.19 | 8.25 | 1.62 | 13.10 | 4.32 | 85.80 | 10.94 |
|  |  | F | 20 | 26.65 | 2.83 | 21.05 | 3.58 | 18.40 | 2.26 | 8.55 | 1.50 | 13.10 | 3.67 | 87.75 | 11.47 |
|  |  |  | 40 | 25.40 | 3.29 | 21.65 | 2.80 | 18.23 | 2.20 | 8.40 | 1.55 | 13.10 | 3.95 | 86.78 | 11.10 |
|  | BE | M | 20 | 24.60 | 2.80 | 22.05 | 3.72 | 18.85 | 2.01 | 8.75 | 1.25 | 13.90 | 4.01 | 88.15 | 12.31 |
|  |  | F | 20 | 24.65 | 3.10 | 20.90 | 4.59 | 17.80 | 2.91 | 7.80 | 1.79 | 13.45 | 4.26 | 84.60 | 14.12 |
|  |  |  | 40 | 24.63 | 2.91 | 21.48 | 4.16 | 18.33 | 2.53 | 8.28 | 1.60 | 13.68 | 4.09 | 86.38 | 13.20 |
|  | Total | M | 60 | 24.70 | 3.07 | 22.28 | 2.57 | 18.38 | 2.26 | 8.60 | 1.32 | 13.98 | 4.53 | 87.95 | 11.36 |
|  |  | F | 60 | 25.85 | 3.24 | 21.38 | 3.51 | 18.25 | 2.49 | 8.28 | 1.60 | 13.40 | 3.98 | 87.17 | 12.06 |
|  |  | Tot | 120 | 25.28 | 3.20 | 21.83 | 3.10 | 18.32 | 2.37 | 8.44 | 1.47 | 13.69 | 4.26 | 87.56 | 11.67 |
